# Fucoidan Ameliorates Renal Injury-Related Calcium-Phosphorus Metabolic Disorder and Bone Abnormality in the CKD–MBD Model Rats by Targeting FGF23-Klotho Signaling Axis

**DOI:** 10.3389/fphar.2020.586725

**Published:** 2021-01-28

**Authors:** Bu-Hui Liu, Fee-Lan Chong, Can-Can Yuan, Ying-Lu Liu, Hai-Ming Yang, Wen-Wen Wang, Qi-Jun Fang, Wei Wu, Mei-Zi Wang, Yue Tu, Zi-Yue Wan, Yi-Gang Wan, Guo-Wen Wu

**Affiliations:** ^1^ Department of Traditional Chinese Medicine, Nanjing Drum Tower Hospital Clinical College of Traditional Chinese and Western Medicine, Nanjing University of Chinese Medicine, Nanjing, China; ^2^ Nephrology Division, Affiliated Hospital of Nanjing University of Chinese Medicine, Nanjing, China; ^3^ The School of Pharmacy, Management and Science University, Shah Alam, Malaysia; ^4^ Department of Traditional Chinese Medicine, Nanjing Drum Tower Hospital, The Affiliated Hospital of Nanjing University Medical School, Nanjing, China; ^5^ Department of Traditional Chinese Medicine Health Preservation, Acupuncture, Moxibustion and Massage College, Health Preservation and Rehabilitation College, Nanjing University of Chinese Medicine, Nanjing, China; ^6^ Department of Social Work, Meiji Gakuin University, Tokyo, Japan; ^7^ Jilin Province Huinan Chonglong Bio-Pharmacy Co., Ltd., Huinan, China

**Keywords:** fucoidan, chronic kidney disease-mineral and bone disorder, FGF23-Klotho signaling axis, phosphorus reabsorption, ERK1/2-SGK1-NHERF-1-NaPi-2a pathway

## Abstract

**Background: **Recently, chronic kidney disease (CKD)-mineral and bone disorder (MBD) has become one of common complications occurring in CKD patients. Therefore, development of a new treatment for CKD–MBD is very important in the clinic. In China, Fucoidan (FPS), a natural compound of *Laminaria japonica* has been frequently used to improve renal dysfunction in CKD. However, it remains elusive whether FPS can ameliorate CKD–MBD. FGF23-Klotho signaling axis is reported to be useful for regulating mineral and bone metabolic disorder in CKD–MBD. This study thereby aimed to clarify therapeutic effects of FPS in the CKD–MBD model rats and its underlying mechanisms *in vivo* and *in vitro*, compared to Calcitriol (CTR).

**Methods: **All male rats were divided into four groups: Sham, CKD–MBD, FPS and CTR. The CKD–MBD rat models were induced by adenine administration and uninephrectomy, and received either FPS or CTR or vehicle after induction of renal injury for 21 days. The changes in parameters related to renal dysfunction and renal tubulointerstitial damage, calcium-phosphorus metabolic disorder and bone lesion were analyzed, respectively. Furthermore, at sacrifice, the kidneys and bone were isolated for histomorphometry, immunohistochemistry and Western blot. *In vitro*, the murine NRK-52E cells were used to investigate regulative actions of FPS or CTR on FGF23-Klotho signaling axis, ERK1/2-SGK1-NHERF-1-NaPi-2a pathway and Klotho deficiency.

**Results: **Using the modified CKD–MBD rat model and the cultured NRK-52E cells, we indicated that FPS and CTR alleviated renal dysfunction and renal tubulointerstitial damage, improved calcium-phosphorus metabolic disorder and bone lesion, and regulated FGF23-Klotho signaling axis and ERK1/2-SGK1-NHERF-1-NaPi-2a pathway in the kidney. In addition, using the shRNA-Klotho plasmid-transfected cells, we also detected, FPS accurately activated ERK1/2-SGK1-NHERF-1-NaPi-2a pathway through Klotho loss reversal.

**Conclusion: **In this study, we emphatically demonstrated that FPS, a natural anti-renal dysfunction drug, similar to CTR, improves renal injury-related calcium-phosphorus metabolic disorder and bone abnormality in the CKD–MBD model rats. More importantly, we firstly found that beneficial effects *in vivo* and *in vitro* of FPS on phosphorus reabsorption are closely associated with regulation of FGF23-Klotho signaling axis and ERK1/2-SGK1-NHERF-1-NaPi-2a pathway in the kidney. This study provided pharmacological evidences that FPS directly contributes to the treatment of CKD–MBD.

## Introduction

In the clinic, chronic kidney disease (CKD) patients with renal dysfunction often suffer from disturbances in mineral and bone disorder including metabolic dysregulation of calcium (Ca^2+^), phosphorus (P^4+^), parathyroid hormone, vitamin D and fibroblast growth factor 23 (FGF23), that ultimately causes ectopic calcification in vasculature and bone abnormality (Hruska et al., 2017). These renal dysfunction-associated complications of CKD patients cover a broader syndrome and are defined as CKD-mineral and bone disorder (CKD–MBD) (Ketteler et al., 2017). Recently, CKD–MBD has become one of common complications occurring in CKD patients at stage G3a to G5D (Liu et al., 2019). Therefore, development of a new treatment for CKD–MBD is very important in the clinic. It is reported that vitamin D agents and vitamin D receptor activators are widely used to control CKD–MBD in CKD patients. Vitamin D is also known to have several pleiotropic actions on bone and mineral metabolism, immune function and cardiovascular systems (Nagpal et al., 2005). Despite this, the effects of vitamin D agents and vitamin D receptors on bone and vasculature are complicated. Here, it should be noted that an excessive administration of vitamin D agents or vitamin D receptors can result in induction of vascular calcification (Rodriguez et al., 2011). Hence, it is now necessary, using the CKD–MBD animal model, to demonstrate potential mechanisms of CKD–MBD and further to achieve appropriate management strategies for CKD–MBD patients.

Bone abnormalities in the patients and animal models with CKD–MBD are characterized by renal osteodystrophy, disorder of mineral metabolism and volume and increased propensity to fracture (Tamagaki et al., 2006; Aleksova et al., 2018; Watanabe et al., 2018; Waziri et al., 2019). In which, calcium-phosphorus metabolic disorder and osteoporosis-like pathological lesions are the early hallmarks in these CKD–MBD patients with advanced renal dysfunction (Aleksova et al., 2018; Waziri et al., 2019). Recently, FGF23 as a phosphate-regulating hormone has been discovered by the studies on rare hypophosphatemic disorder, whereas Klotho, which subsequently turns out to be a co-receptor for FGF23, is identified in a mouse model showing hyperphosphatemia and multiple aging-like traits. Briefly, FGF23-Klotho signaling axis is a pivotal regulator of mineral and bone metabolic disorder in CKD–MBD (Olauson et al., 2014). In addition, the inchoate onset of Klotho deficiency contributes to FGF23 resistance in the kidney and a maladaptive increase in circulating FGF23. FGF23 is also an early biomarker of renal injury and increased FGF23 predicts adverse clinical outcomes, in particular cardiovascular complications of CKD–MBD (Moe et al., 2009; Erben and Andrukhova, 2017; Kuro-O, 2017). Consequently, targeting FGF23-Klotho signaling axis in response to renal injury and its correlative mineral and bone metabolic disorder has been identified as multi-targets in the treatment of CKD–MBD.

Fucoidan (FPS) is a class of fucose-rich sulfated carbohydrates found in brown marine algae and echinoderms, and more recently identified in *Laminaria japonica*, a traditional Chinese herbal medicine (van Weelden et al., 2019). Previous studies showed that FPS has an attractive array of bioactivities and potential applications including immune modulation, cancer inhibition and pathogen inhibition (Fitton et al., 2015). Over the last few years, some researches into FPS have continued to gain pace and point toward potential therapeutic or adjunct roles in CKD and osteoporosis. Wang et al. reported that a modern preparation of FPS, Haikun Shenxi capsule (HKSX, the local name in China) approved by the China State Food and Drug Administration for the treatment of CKD (Z20030052) has ameliorative effects on chronic renal failure (CRF), acute kidney injury (AKI) and diabetic kidney disease (DKD) in the clinic (Wang et al., 2019). Besides, more importantly, Hwang et al. and Jin et al. consecutively found that the low-molecular weight FPS also inhibits differentiation of osteoclasts and reduces osteoporosis *in vivo* and *in vitro* (Hwang et al., 2016; Jin et al., 2017). It thereby seems reasonable to hypothesize that aiming at renal injury and bone abnormality, FPS may provide a new and appropriate therapeutic approach for CKD–MBD patients. However, so far, there are still some important issues unresolved in the role of renal injury and its correlative bone abnormality in CKD–MBD treated by FPS, for instance, whether FPS can improve renal injury and bone abnormality, and if so, what are underlying mechanisms involved *in vivo* and *in vitro*.

Here, to address these issues, we designed continuous and correlative animal and cell experiments to verify the hypotheses that FPS might ameliorate renal injury-related calcium-phosphorus metabolic disorder and bone abnormality in CKD–MBD by targeting FGF23-Klotho signaling axis and its downstream pathway, compared to Calcitriol (1α,25-dihydroxyvitamin D_3_, CTR). The results in agreement with these hypotheses will suggest that regulation of FGF23-Klotho signaling axis is protective in CKD–MBD.

## Materials and Methods

### Animals, Drugs and Reagents

All experiments were performed using the male Sprague–Dawley rats weighing from 200 to 220 g, purchased from the Experimental Animal Center of Nanjing University (Nanjing, China) [License No: SCXK (Shanghai) 2012-0006]. All rats were housed (six rats/cage) at 22 ± 3°C and 50 ± 10% humidity using a 12-hour light/dark cycle, and were fed a specific-pathogen free (SPF)-grade standard rat chow (catalog number 1010008) from Xietong Pharmaceutical Bio-engineering Co., Ltd. (Nanjing, China), and were given tap water ad libitum in the Experiment Animal Center of Nanjing Drum Tower Hospital, The Affiliated Hospital of Nanjing University Medical School. Surgical procedures and experimental protocols were approved by the Animal Ethics Committee of Nanjing University Medical School. Adenine was purchased from Sigma-Aldrich (St. Louis, MO, United States) and solubilized in distilled water and administrated via gavage individually. FPS (C_6_H_10_O_7_S, CAS: 9072-19-9) was obtained from Jilin Province Huinan Chonglong Bio-Pharmacy Co., Ltd (Huinan, China). FPS was dissolved in 10% dimethyl sulfoxide (DMSO) with a termination concentration of 1 g/L. CTR was obtained from Cayman Chemical Company, Inc (Ann Arbor, MI). CTR was dissolved in 10% DMSO with a termination concentration of 10^−5^ mol/L. Antibody against Klotho was purchased from Abcam Technology (Abcam, Cambridge, MA), antibodies against FGF23, FGF receptor1 (FGFR1), phosphorylated extracellular signal-regulated kinase (ERK)1/2 (*p*-ERK1/2), serum/glucocorticoid-regulated kinase1 (SGK1), Na^+^/H^+^ exchange regulatory cofactor-1 (NHERF-1), sodium-phosphate co-transporter-2a (NaPi-2a) and glyceraldehyde-3-phosphate dehydrogenase (GAPDH) were purchased from Cell Signaling Technology (Beverly, MA, Unites States).

### Animal Experimental Protocols

As shown in Supplemental Figure 1, 28 rats were randomly divided into four groups according to the random number table, seven rats in the sham-operated group (the Sham group), seven rats in the CKD–MBD model group (the CKD–MBD group), seven rats in the FPS-treated group (the FPS group) and seven rats in the CTR-treated group (the CTR group). According to previous studies (Tamagaki et al., 2006; Zhang et al., 2017; Ma and He, 2018; Neven et al., 2018), the model rats with CKD–MBD were induced by adenine administration and uninephrectomy. More specifically, the rats were firstly undergone the left uninephrectomy on day 7, and then, administrated with 2% adenine (150 mg/kg/d) from day 8 to day 21 at the beginning of the experiment. In the sham-operated rats, the left kidney was only exposed during operation. Following administration and operation, the suspensions of FPS (880 mg/kg) and CTR (30 ng/kg) were given to the CKD–MBD model rats, respectively, by daily, morning gastric gavage for 3 weeks, while the sham-operated rats and the CKD–MBD model rats were intervened with 2 ml distilled water (vehicle). During the drug-intervention, every 3 days, the CKD–MBD model rats, the FPS-treated rats and the CTR-treated rats were given 2% adenine at a dose of 150 mg/kg/d to avoid a quick recovery of renal dysfunction. At the end of 3 weeks after administration, all rats were anesthetized by intraperitoneal injection of ketamine and sacrificed via cardiac puncture. The samples of urine and blood, kidneys and the femur bone were collected for detection of various indicators. Specifically, blood and urine samples were centrifuged at 3,000 rpm at 4°C for 10 min to collect serum or supernatant, which were sent to the Department of Laboratory Medicine of Nanjing Drum Tower Hospital, the Affiliated Hospital of Nanjing University Medical School for biochemical analysis. In addition, some serums were frozen at −80°C for enzyme-linked immunosorbent assay. Renal cortex tissues were dissected and frozen at −80°C for Western blotting (WB) analysis, as well as for immunohistochemistry and histopathological staining. The femur bone samples were collected and stored in 4% paraformaldehyde solution at 4°C for further detection. Here, it should be noted that, in the clinic, FPS at a dose of 600 mg/d and CTR at a dose of 0.5 μg/d are used to treat a CRF patient weighing 60 kg, respectively (Liu et al., 2019; Wang et al., 2019). Based on the animal standard conversion formula, the effective amount of FPS or CTR in a rat (200 g) is equivalent to 120 mg/kg/d or 30 ng/kg/d. According to our preliminary experiment, we chose the 880 mg/kg/d dose of FPS in this experiment. In addition, one rat died in each group due to misoperation during the experiment. Therefore, there were 6 rats in each group at the end of the experiment.

### Rats’ Biochemical Parameters

Body weight (BW) and urine protein of the rats were detected, respectively before and every 1 or 2 weeks after induction of renal injury. The right kidneys of the rats in each group were removed and weighed after a cardiac puncture. Kidney hypertrophy index (KHI) was calculated according to the method described by Lane et al. (1992), that is KHI = kidney weight (KW)/BW. At the end of week 3 after the drug-intervention, the rats were anesthetized and blood samples (5 ml) were drawn from the heart. The biochemical parameters in blood including uric acid (UA), serum creatinine (Scr), blood urea nitrogen (BUN), Ca^2+^, P^4+^, alkaline phosphatase (ALP), 25-hydroxyvitamin D_3_ (VD_3_), intact parathyroid hormone (iPTH), alanine aminotransferase (ALT) and aspartate aminotransferase (AST) were tested, respectively. Serum and urine samples were measured using an automatic biochemical analyzer (Beckman Instruments, Inc., California, United States) in the Department of Laboratory Medicine of Nanjing Drum Tower Hospital, The Affiliated Hospital of Nanjing University Medical School.

### Rats’ Bone Mineral Density and Micro-CT Analysis

The lumbar vertebrae and femur bone of each rat were scanned using the dual-energy X-ray absorptiometry (GE, USA). Their bone mineral density (BMD) as the total bone mineral density (TBBMD) and the femur bone mineral density (FBBMD) were measured, respectively as described by Martin and Gutman (1978). Micro-CT analysis structural properties of the femur bone were determined with a high-resolution micro-CT system (Hiscan M-1001 *in-vivo* Micro-CT System). Moist bones were wrapped in parafilm and covered with dental wax to prevent drying and movement during scanning. The X-ray tube was set to 60 kV, and the beam was filtered with a 0.5 mm aluminium filter. The sample position and camera settings were adjusted to provide a 3.0 μm isotropic pixel size, and projection images were collected every 0.2°. Tissue mineral density values were calibrated against hydroxyapatite phantoms with densities of 250 and 750 mg/cm^3^. Reconstructions were done with NRecon (v 1.6.9.8; Bruker micro-CT), where appropriate corrections to reduce beam hardening and ring artefacts were applied. Bone was segmented in slices of 3 μm thickness. After 600 slices from the growth plate, we selected and analysed 120 slices of trabecular bone. Analyses were performed in agreement with guidelines for assessment of bone microstructure in rodents using micro-CT (Bouxsein et al., 2010). Trabecular bone morphology was analyzed by applying global threshold and despeckle to provide binary image for three-dimensional analyses. The parameters including total tissue volume (TV), bone volume (BV), bone volume/tissue volume ratio (BV/TV), trabecular number (Tb.N), trabecular bone separation (Tb.Sp), trabecular bone thickness (Tb.Th) and average CT value were obtained.

### Light Microscopy Examination

The tissue samples from renal cortex for light microscopy (LM) assessment were fixed with 10% neutral buffered formalin, embedded in paraffin, cut into 3 μm-thick sections and stained with periodic acid-Schiff (PAS) or Masson reagent. Similarly, the tissue samples of the femur bone were also cut into 10 μm-thick sections and stained with Hematoxylin-Eosin (HE). Semiquantitative morphological studies of renal injury were carried out by randomly selecting 20 full-sized tubulointerstitial areas from each specimen. The relative area of ECM (score) in tubulointerstitium and tubulointerstitial collagen deposition (score) were calculated with Image-Pro Plus 6.0 software (Media Cybernetic). The results were confirmed by the pathological professional doctor.

### Immunohistochemistry Assay

Klotho, FGF23 and FGFR1 were detected in 3 μm-thick paraffin-embedded renal sections. For immunohistochemistry (IHC) staining of Klotho, FGF23 and FGFR1 antibodies against-Klotho, FGF23 and FGFR1 (Serotec, Oxford, United Kingdom) were used, respectively. Quantitative analysis of Klotho, FGF23 and FGFR1 was performed in a blinded fashion and expressed as cells/tubulointerstitial cross-section. The results were confirmed by the pathological professional doctor.

### Enzyme-Linked Immunosorbent Assay

Enzyme-linked immunosorbent assays for assaying the level of serum FGF23 according to manufacturer’s instructions (R&D Systems Inc., Minneapolis, MN, Unites States).

### Western Blotting Analysis *in Vivo*

WB analysis *in vivo* was performed as previously described (Wu et al., 2018; Han et al., 2019; Liu et al., 2019). The protein expressions of Klotho, FGF23, FGFR1, *p*-ERK, *p*-SGK1, NHERF-1, NaPi-2a and GAPDH were examined, respectively and quantitated by Densitograph (ATTO). The data were shown as ratios relative to control findings and expressed as mean ± standard deviation (S.D.) of 3 independent experiments.

### Renal Proximal Tubular Epithelial Cell Culture and Treatment

The NRK-52E cells, a rat renal proximal tubular epithelial cell line, were cultured in Dulbecco’s modified Eagle’s medium/Ham’s F-12 (HyClone) supplemented with 5% fetal bovine serum (FBS; Gibco, Grand Island, NY) as described previously (Tu et al., 2017). The NRK-52E cells were exposed to transforming growth factor-β (TGF-β, 5 ng/ml) and recombinant human FGF23 R176/179Q (rFGF23, 100 ng/ml) without or with FPS at the dose of 20 μg/ml or CTR at the dose of 10^−7^ mol/L for 24 h. Here, the doses of FPS and CTR were determined by the references of Jia et al. (2016) and Lin et al. (2016).

### Cell Viability Assessment

The NRK-52E cell viability was assessed using CCK-8 (Beyotime, Shanghai, China). The cells were seeded into 96-well plates, with 3 replicate wells for each group, at a density of 1 × 10^4^ cells per well, with 100 μl medium. After the cells were incubated for the indicated time, 10 μl of the CCK-8 solution was added to each well, followed by incubation for 2 h. The optical density (OD) was computed at the absorbance of 450 nm, and the cell viability was calculated.

### Lentiviral Transfection

Lentiviral vectors expressing non-targeting pLKO.1 controlled shRNA, and shRNA constructs targeting shRNA-Klotho were obtained from Sigma-Aldrich. The NRK-52E cells were transfected with shRNA plasmid with Lipofectamine 2000 (Invitrogen) following the manufacturer’s instructions. The infected NRK-52E cells were then incubated for 24 h. After the lentiviral transfection, the lentivirus-containing medium was removed and replaced with the fresh growth medium. The infected NRK-52E cells were cultured for another 48 h, and the medium was changed every 24 h.

### Western Blot Analysis *in Vitro*

The NRK-52E cells were treated in the different groups for 24 h, respectively. After the treatment, cell lysates were separated by gel electrophoresis and blotted with antibodies against Klotho, FGFR1, *p*-ERK, *p*-SGK1, NaPi-2a and GAPDH. The secondary antibody was HRP-conjugated anti-rabbit IgG antibody. WB analysis for cells was carried out according to our previous protocols (Tu et al., 2017; Liu et al., 2018).

### Statistics Analysis

The differences among groups were analyzed by one-way analysis of variance (ANOVA), and the least significant difference (LSD) method was used for multiple comparisons. Qualitative data were analyzed using Fisher’s exact test as indicated. *p <* 0.05 was considered statistically significant.

## Results

### The *in Vivo* Investigations on Characteristics of Renal Injury and Bone Abnormality

**FIGURE 1:**
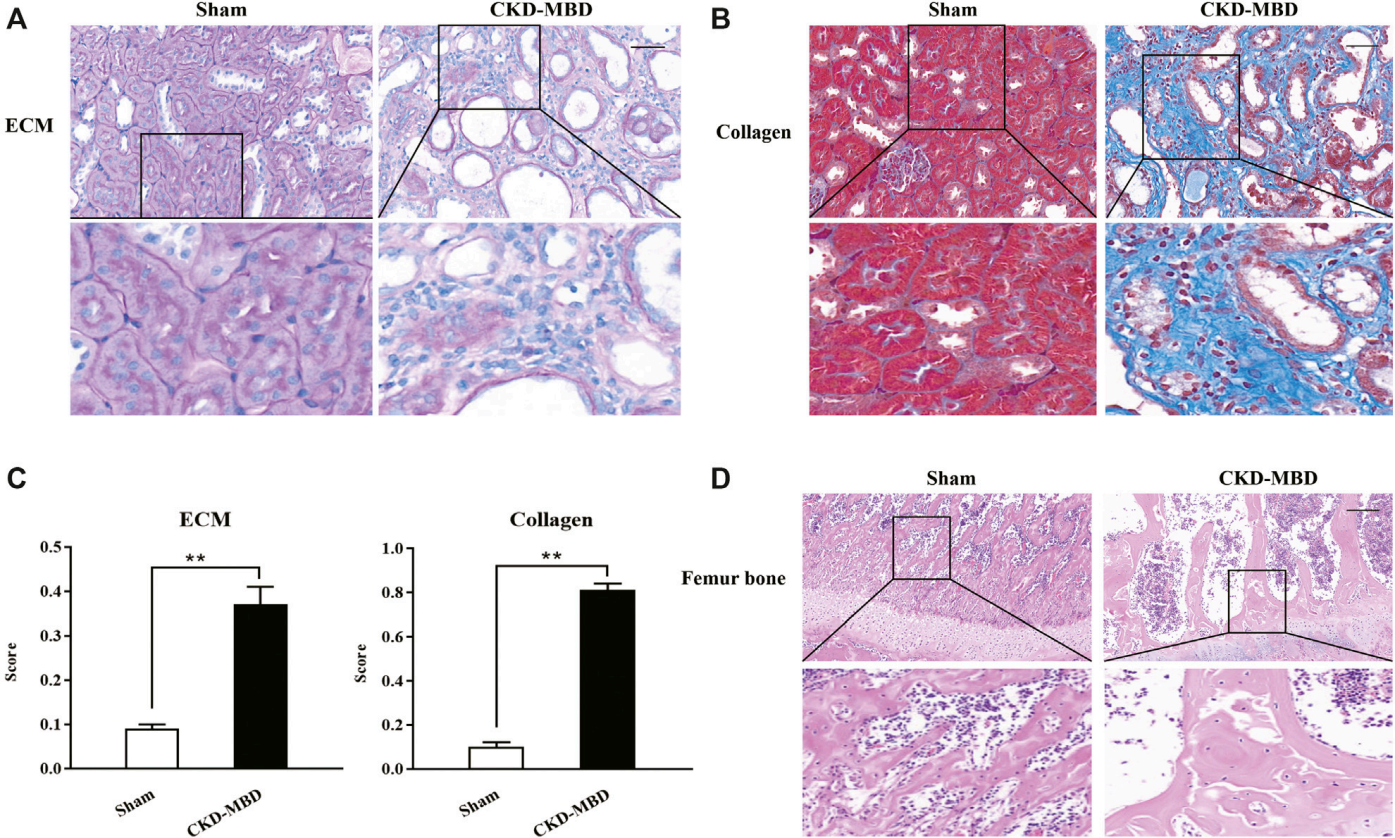
Histopathological characteristics in renal tubulointerstitium and the femur bone between the sham-operated rats and the CKD–MBD model rats **(A)** ECM in renal tubulointerstitium, PAS staining × 200 **(B)** Collagen in renal tubulointerstitium, Masson staining × 200 **(C)** Scores of ECM and Collagen in renal tubulointerstitium **(D)** Pathological lesions in the femur bone, HE staining × 200. Scale bar = 100 μm. The data are expressed as mean ± S.D. **p < 0.01.

Firstly, using the modified CKD–MBD rat models induced by adenine administration and uninephrectomy, we observed KW, BW, KHI, proteinuria and renal functional indicators including UA, Scr and BUN, compared to the sham-operated rats. As shown in Table 1, in addition to BW, the increase in values of KW, KHI, proteinuria, UA, Scr and BUN was observed significantly at 3 weeks after induction of renal injury, compared to those of the sham-operated rats. Meantime, PAS and Masson staining showed that consecutive adenine-administrations to the rats with a safe and effective dose of 150 mg/kg/d resulted in significant renal tubulointerstitial damages including tubular atrophy, interstitial cells infiltration, ECM accumulation and collagen deposition in tubulointerstitial area at 3 weeks after induction of renal injury (Figures 1A,B). Here, obvious increased scores of ECM and collagen in tubulointerstitial area of the kidneys in the CKD–MBD model rats were detected, respectively, compared to those of the sham-operated rats (Figure 1C).

**TABLE 1:** The comparison of characteristics of renal injury and bone abnormality between the sham-operated rats and the CKD–MBD model rats.

	The sham-operated rats	The CKD–MBD model rats
BW (g)	417 ± 7.51	343 ± 2.00**
KW (g)	1.63 ± 0.29	3.55 ± 0.67**
KHI (g/kg)	3.87 ± 0.72	10.86 ± 2.08**
Serum		
UA (μmol/L)	73 ± 25.24	227 ± 110.49*
Scr (μmol/L)	22.67 ± 2.52	175 ± 4.00**
BUN (mmol/L)	8.53 ± 0.12	38.6 ± 0.79**
Ca^2+^ (mmol/L)	3.45 ± 0.18	2.23 ± 0.06**
P^4+^ (mmol/L)	2.32 ± 0.17	3.62 ± 0.14**
ALP (U/L)	131.17 ± 6.69	257.70 ± 9.13**
VD_3_ (ng/ml)	28.78 ± 1.75	16.64 ± 2.45**
FGF23 (ng/L)	909.30 ± 75.25	1210.00 ± 82.38**
iPTH (pg/ml)	9.61 ± 0.62	19.23 ± 1.7**
Urine		
Ca^2+^ (mmol/L)	0.76 ± 0.01	2.14 ± 0.08**
P^4+^ (mmol/L)	18.41 ± 0.44	16.26 ± 0.06**
Pro (g/24 h)	3.13 ± 0.39	18.69 ± 2.34**
TBBMD (g/cm^2^)	0.17 ± 0.01	0.15 ± 0.01**
FBBMD (g/cm^2^)	0.19 ± 0.01	0.15 ± 0.01**

The data are expressed as mean ± S.D.

*p < 0.05, **p < 0.01 vs. the sham-operated rats.

Next, we examined the indexes of urine Ca^2+^ and P^4+^, serum Ca^2+^, P^4+^, ALP, VD_3_, FGF23 and iPTH, as well as TBBMD, FBBMD and pathological lesions of the femur bone in the sham-operated rats and the CKD–MBD model rats. Also as shown in Table 1, significant changes of urine Ca^2+^ and P^4+^, serum Ca^2+^, P^4+^, ALP, VD_3_, FGF23 and iPTH, as well as obvious decline in TBBMD and FBBMD were found in the CKD–MBD model rats, respectively, compared to those of the sham-operated rats. Furthermore, typical bone lesions with resorption cavities in the cortical bone were clearly revealed in the CKD–MBD model rats at 3 weeks after induction of renal injury as shown by HE staining. Notably, pathological lesions of the femur bone in the CKD–MBD model rats were characterized by an increase in osteoid and resorption cavities with osteoclasts, osteoblasts and fibrosis in the cortical bone (Figure 1D).

These results indicated that renal injury and bone abnormality in the CKD–MBD model rats were both revealed significantly *in vivo*.

### The *in Vivo* Effects of FPS and CTR on Renal Dysfunction and Renal Tubulointerstitial Damage

**FIGURE 2:**
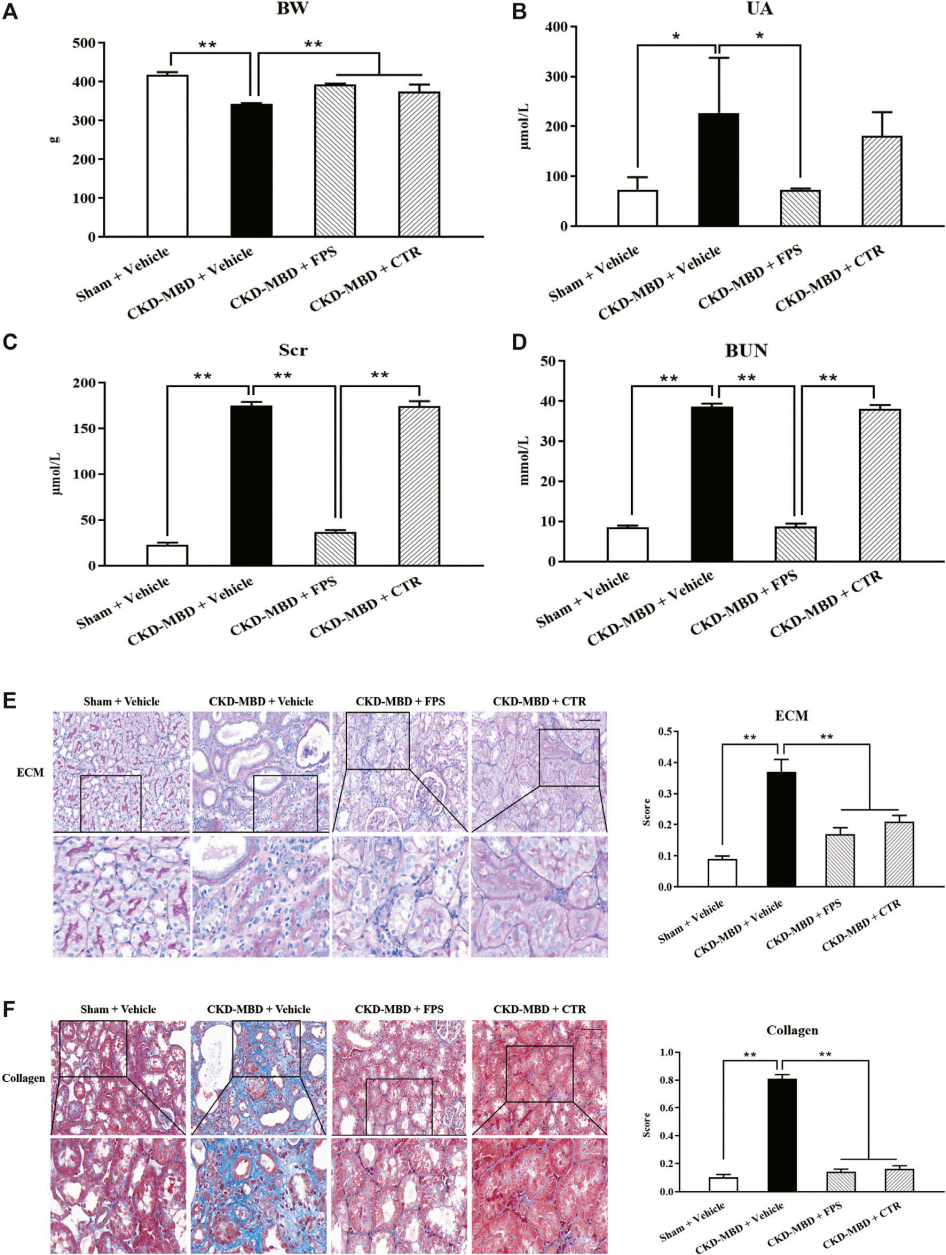
FPS and CTR alleviated renal dysfunction and renal tubulointerstitial damage in the CKD–MBD model rats **(A)** BW **(B)** UA **(C)** Scr **(D)** BUN **(E)** ECM and its score in renal tubulointerstitium, PAS staining × 200 **(F)** Collagen and its score in renal tubulointerstitium, Masson staining × 200. Scale bar = 100 μm. The data are expressed as mean ± S.D. *p < 0.05, **p < 0.01.

We checked the effects of FPS and CTR on renal dysfunction in the CKD–MBD model rats. After induction of renal injury, the rats in the CKD–MBD, FPS and CTR groups showed BW loss in different degrees. Thereinto, the changes of the CKD–MBD group rats were particularly obvious. Compared with the Sham group rats, BW of the CKD–MBD group rats rose slowly. At the end of 3 weeks after the treatment of FPS or CTR, BW of the FPS and CTR groups rats was increased even faster, compared to that of the CKD–MBD group rats (Figure 2A). Moreover, the levels of UA, Scr and BUN in the CKD–MBD group rats were increased significantly, compared to those of the Sham group rats. After the treatment of FPS for 3 weeks, the levels of UA, Scr and BUN in the CKD–MBD model rats were decreased significantly, compared to those of the CKD–MBD group rats. By contrast, after the treatment of CTR for 3 weeks, the levels of UA, Scr and BUN in the CKD–MBD model rats were not changed obviously. Here, it’s worth noting that the actions of FPS improving Scr and BUN were better than CTR (Figures 2B–D).

Then, we confirmed the effects of FPS and CTR on renal tubulointerstitial damage in the CKD–MBD model rats. As shown in Figure 2, at the end of 3 weeks after induction of renal injury, upon the observation under LM, entire and clear renal interstitium and open renal tubules were detected in the Sham group rats. By comparison, obvious renal tubulointerstitial damages such as ECM accumulation and collagen deposition in tubulointerstitial area of the CKD–MBD group rats were found, respectively, compared to those of the Sham group rats. After the treatment of FPS or CTR for 3 weeks, ECM accumulation and collagen deposition in tubulointerstitial area of the FPS and CTR groups rats were improved significantly, compared to those of the CKD–MBD group rats (Figures 2E,F).

These results indicated that renal dysfunction and renal tubulointerstitial damage in the CKD–MBD model rats could be alleviated by FPS and CTR *in vivo*.

### The *in Vivo* Effects of FPS and CTR on Calcium-Phosphorus Metabolic Disorder and Bone Lesion

**FIGURE 3:**
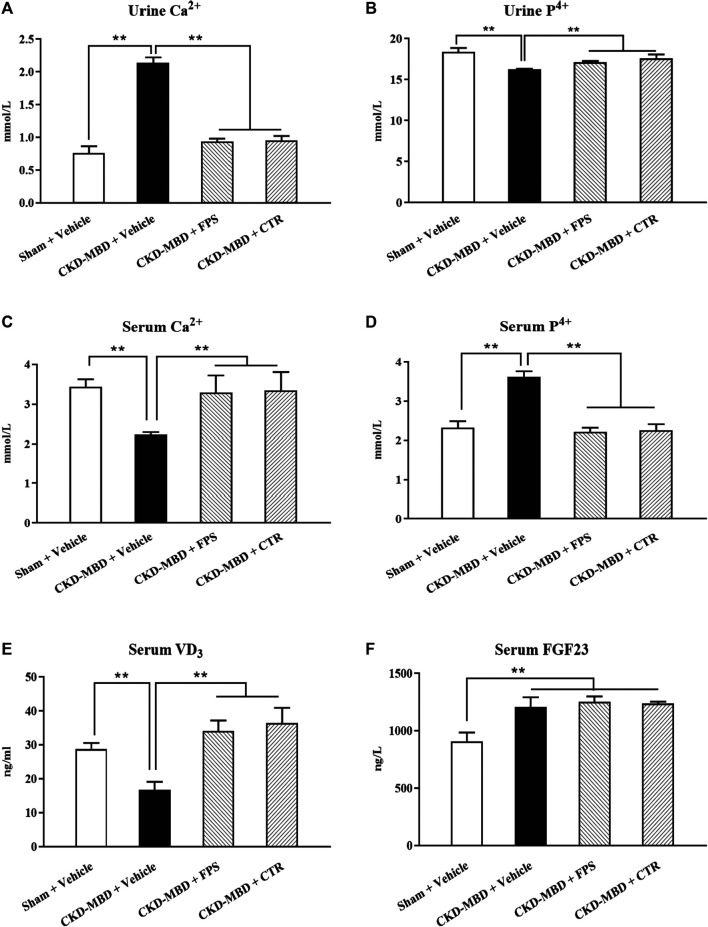
FPS and CTR improved calcium-phosphorus metabolic disorder in the CKD–MBD model rats **(A)** Urine Ca^2+^ **(B)** Urine P^4+^ **(C)** Serum Ca^2+^ **(D)** Serum P^4+^ **(E)** Serum VD_3_ **(F)** Serum FGF23. The data are expressed as mean ± S.D. **p < 0.01.

**FIGURE 4:**
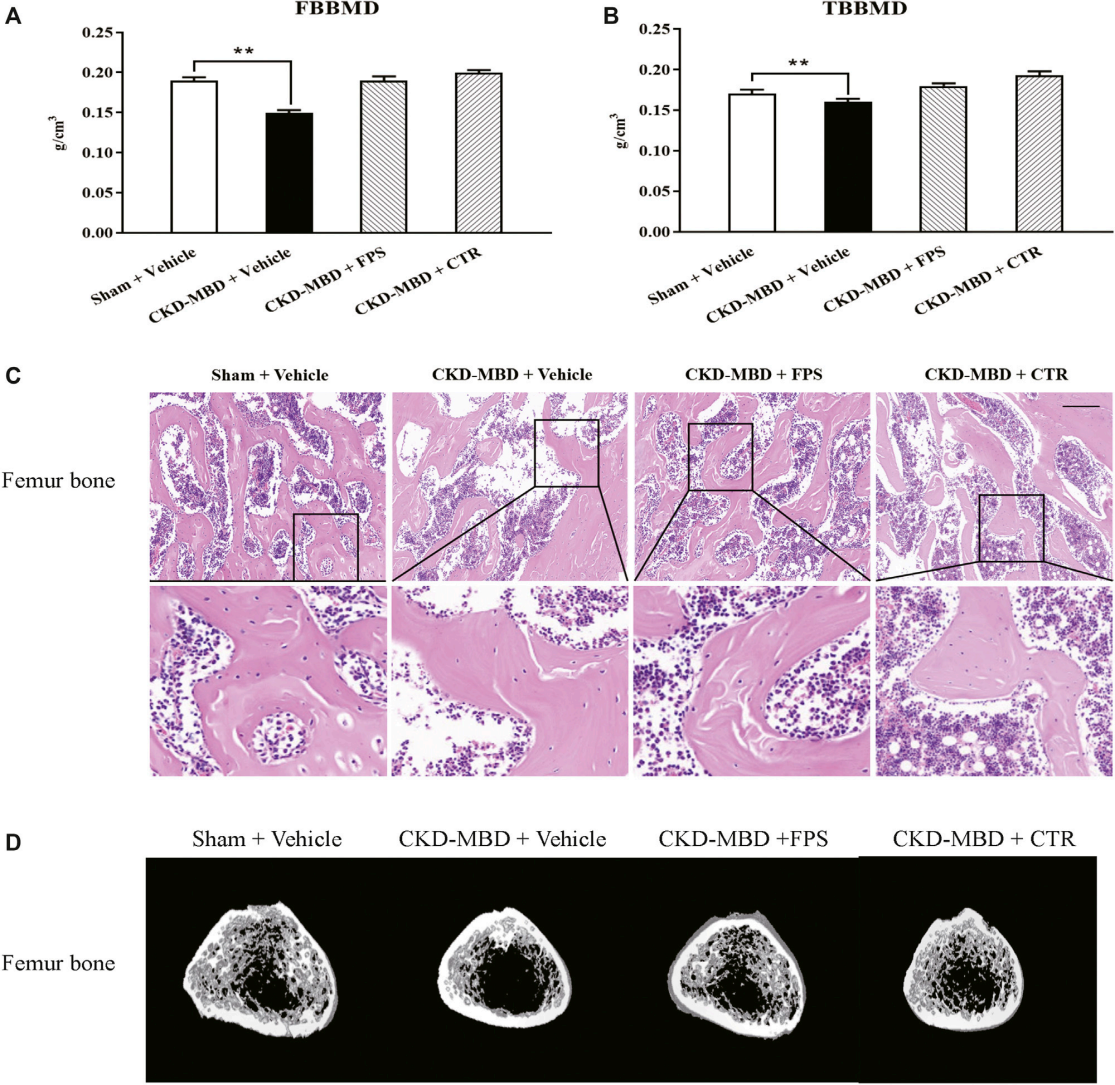
FPS and CTR improved bone lesion in the CKD–MBD model rats **(A)** FBBMD **(B)** TBBMD **(C)** Pathological lesions in the femur bone, HE staining × 200 **(D)** Images acquired by micro-CT. Scale bar = 100 μm. The data are expressed as mean ± S.D. **p < 0.01.

We evaluated the actions of FPS and CTR on the levels of urine Ca^2+^ and P^4+^, serum Ca^2+^, P^4+^, VD_3_ and FGF23, as well as BMD in the CKD–MBD model rats. As shown in Figures 3, 4, after induction of renal injury, in line with renal dysfunction, increased levels of urine Ca^2+^, serum P^4+^ and FGF23, decreased levels of urine P^4+^, serum Ca^2+^ and VD_3_, as well as reduced value of BMD in the femur bone and lumbar vertebrae of the CKD–MBD model rats were revealed, respectively, compared to those of the Sham group rats. After the treatment of FPS or CTR for 3 weeks, in addition to serum FGF23 and BMD, the levels of urine Ca^2+^, P^4+^, serum Ca^2+^, P^4+^ and VD_3_ in the CKD–MBD model rats were ameliorated significantly, compared to those of the CKD–MBD group rats (Figures 3A–E and Figures 4A,B).

Next, we verified the effects of FPS and CTR on pathological lesions of the femur bone in the CKD–MBD model rats using histopathological observation and micro-CT analysis. As shown in Figures 4C,D, compared with the CKD–MBD group rats, osteoporosis-like pathological lesions in the femur bone of the CKD–MBD model rats including thinner and deranged trabeculae with increased lacuna were slightly reversed by the treatment of FPS or CTR. Accordingly, the micro-CT analysis also showed that the values of Tb.Th, Tb.Sp, TV, BV, BV/TV, Tb.N and average CT value were ameliorated at different levels in the CKD–MBD model rats after the treatment of FPS or CTR, compared to those of the CKD–MBD group (Table 2).

**TABLE 2:** The comparison of micro-CT parameters in trabecular bone among the four group rats.

Parameters	The Sham group	The CKD–MBD group	The FPS group	The CTR group
TV (mm^3^)	79.92 ± 4.91	64.90 ± 3.2^&&^	76.07 ± 4.75^#^	76.49 ± 7.14^#^
BV (mm^3^)	34.13 ± 6.04	17.54 ± 2.78^&&^	28.91 ± 3.27^#^	27.47 ± 3.77^#^
BV/TV	0.43 ± 0.06	0.27 ± 0.06*	0.38 ± 0.05^#^	0.35 ± 0.06
Tb.Th (mm)	0.52 ± 0.06	0.4 ± 0.03^&&^	0.5 ± 0.03^#^	0.48 ± 0.03^#^
Tb.Sp (mm)	0.54 ± 0.04	1.00 ± 0.14^&&^	0.72 ± 0.16^#^	0.68 ± 0.14^#^
Tb.N (mm^−1^)	1.21 ± 0.13	1.01 ± 0.08*	1.46 ± 0.11^@@^	1.24 ± 0.19^#^
Average CT value (HU)	2990.6 ± 75.83	2506.67 ± 50.48^&&^	2784.66 ± 71.71^@@^	2688.5 ± 101.09^#^

The data are expressed as mean ± S.D. *p < 0.05, ^&&^p < 0.01 vs. the Sham group; ^#^p < 0.05, ^@@^p < 0.01 vs. the CKD–MBD group.

These results indicated that calcium-phosphorus metabolic disorder and bone lesion in the CKD–MBD model rats could be improved by FPS and CTR *in vivo*.

### The *in Vivo* and *in Vitro* Effects of FPS and CTR on FGF23-Klotho Signaling Axis in the Kidney

**FIGURE 5:**
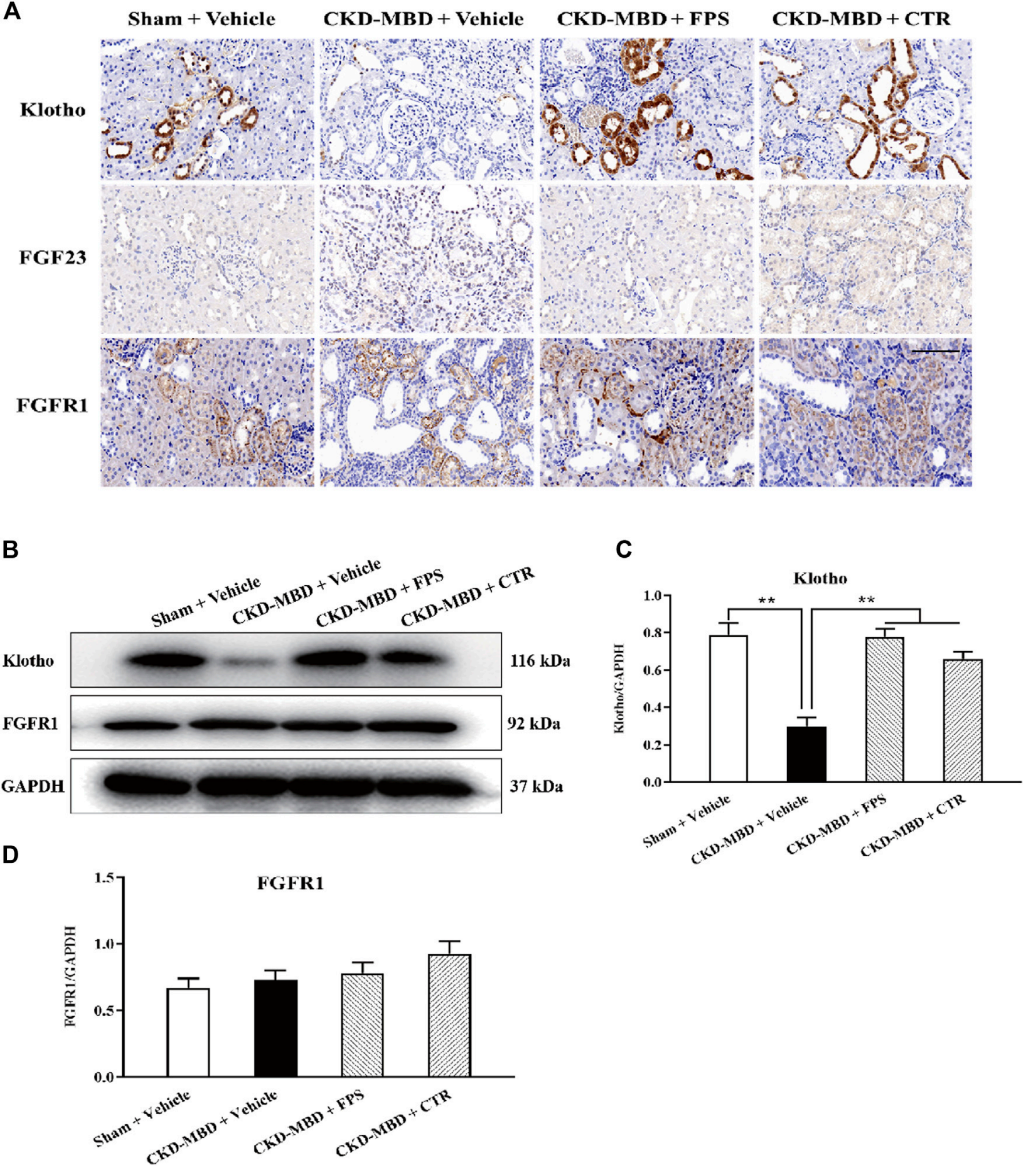
FPS and CTR regulated FGF23-Klotho signaling axis in vivo **(A)** The protein expressional characteristics of Klotho, FGF23 and FGFR1 in renal tubulointerstitium of the kidneys in the four group rats. IHC staining × 200. Scale bar 100 µm **(B)** The protein expressional levels of Klotho, FGF23 and FGFR1 in the kidney of the four group rats **(C)** The rate of Klotho/GAPDH (D) The rate of FGF23/GAPDH **(E)** The rate of FGFR1/GAPDH. The data are expressed as mean ± S.D. **p < 0.01.

We investigated the actions of FPS and CTR on the expressions of critical signaling molecules in FGF23-Klotho signaling axis including Klotho, FGF23 and FGFR1 in renal tubulointerstitium and the kidneys of the CKD–MBD model rats by IHC staining and WB analysis. As shown in Figures 5A,B, after induction of renal injury, in addition to FGF23, decreased IHC staining extents of Klotho and FGFR1 in renal tubulointerstitium of the CKD–MBD model rats were detected obviously. Meanwhile, decreased protein expression level of Klotho and increased protein expression level of FGFR1 in the kidneys were revealed significantly, compared to those of the Sham group rats. After the treatment of FPS or CTR for 3 weeks, the expression of Klotho, not FGFR1, in renal tubulointerstitium and in the kidneys of the CKD–MBD model rats was ameliorated, respectively, compared to that of the CKD–MBD group rats (Figures 5C,D).

**FIGURE 6:**
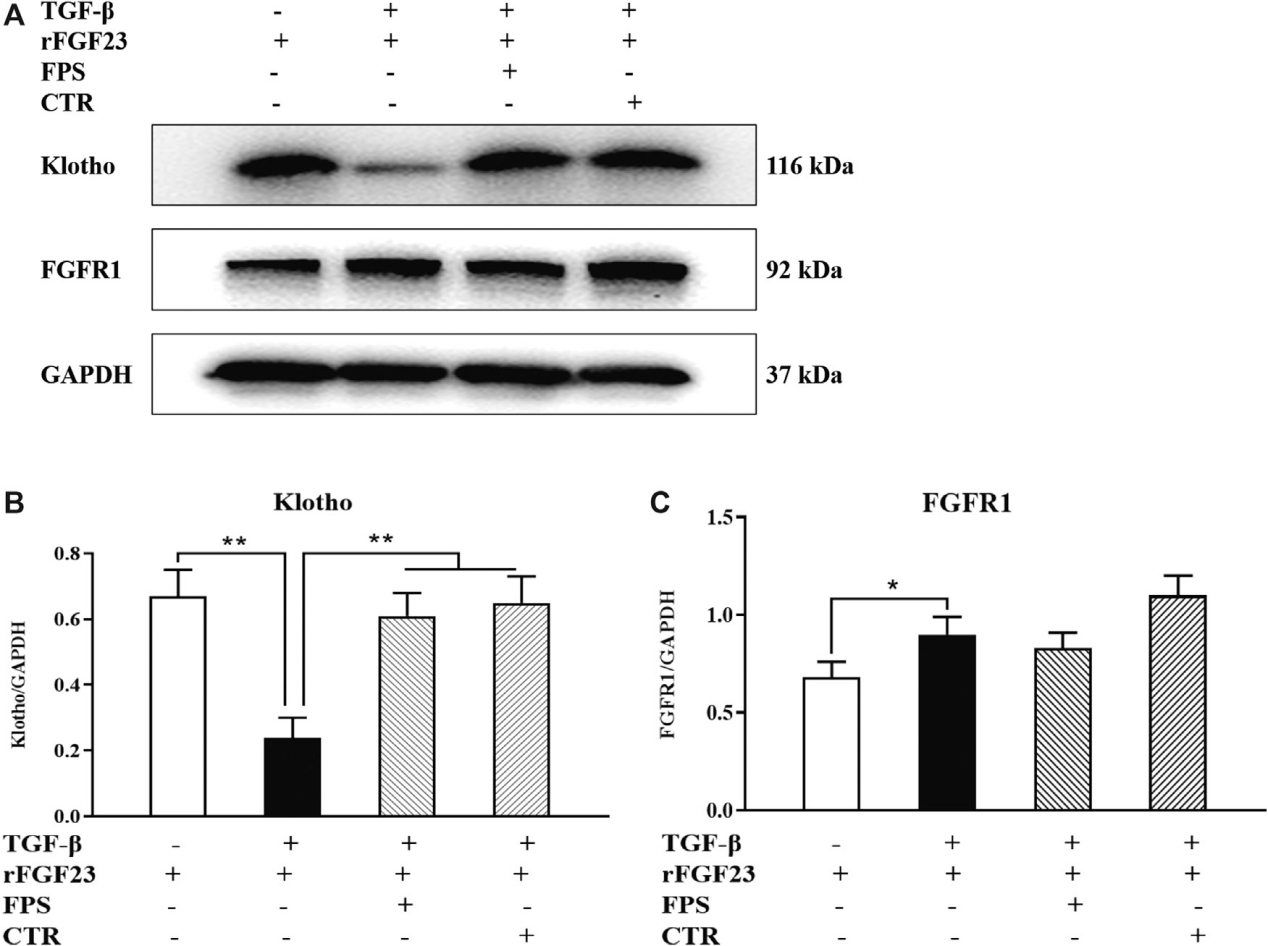
FPS and CTR regulated FGF23-Klotho signaling axis *in vitro* **(A)** The protein expressional levels of Klotho and FGFR1 in the cultured NRK-52E cells exposed to TGF-β and rFGF23 **(B)** The rate of Klotho/GAPDH **(C)** The rate of FGFR1/GAPDH. The data are expressed as mean ± S.D. *p < 0.05, **p < 0.01.

Further, to identify whether FPS and CTR could affect the expressions of critical signaling molecules in FGF23-Klotho signaling axis *in vitro*, we tested the protein expression levels of Klotho and FGFR1 in the cultured NRK-52E cells exposed to TGF-β and rFGF23 for 24 h, compared with the control cells. Prior to the formal cellular experiments, the cytotoxicity of FPS or CTR on the cultured NRK-52E cells was analyzed using CCK-8. As shown in Supplemental Figure 2, the cellular viabilities were significantly decreased under the highest concentrations of FPS at 25 μg/ml (Supplemental Figure 2A) and CTR at 10^−6^ mol/L (Supplemental Figure 2B) compared to the 20 μg/ml dose of FPS and the 10^−7^ mol/L dose of CTR, respectively. Based on these results, the safe and effective doses of FPS (20 μg/ml) and CTR (10^−7^ mol/L) were selected, respectively. In Figure 6, our results showed that rFGF23 decreased Klotho protein expression and increased FGFR1 protein expression in the cultured NRK-52E cells exposed to TGF-β for 24 h, compared to those of the control cells (Figure 6A). In addition, it is noted that the treatment with CTR or FPS at the suitable doses for 24 h significantly improved rFGF23-induced changes in Klotho and FGFR1 protein expressions in the cultured NRK-52E cells exposed to TGF-β, compared to the treatment with rFGF23 (Figures 6B,C). In which, ameliorative effects of FPS and CTR on the protein expression level of Klotho were obvious (Figure 6B).

These results indicated that FGF23-Klotho signaling axis in the kidney could be regulated by FPS and CTR *in vivo* and *in vitro*.

### The *in Vivo* and *in Vitro* Effects of FPS and CTR on ERK1/2-SGK1-NHERF-1-NaPi-2a Pathway in the Kidney

**FIGURE 7:**
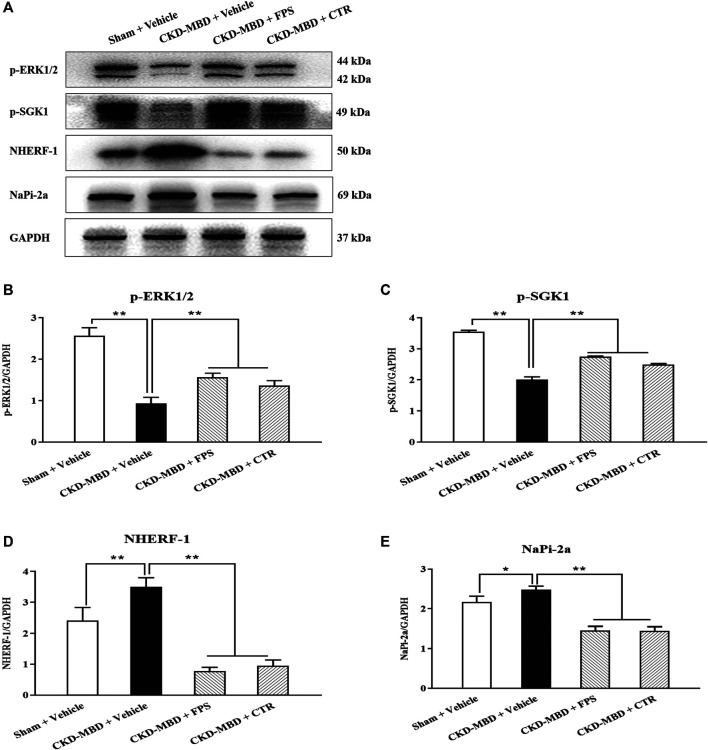
FPS and CTR adjusted ERK1/2-SGK1-NHERF-1-NaPi-2a pathway *in vivo* **(A)** The protein expressional levels of *p*-ERK1/2, *p*-SGK1, NHERF-1 and NaPi-2a in the kidneys of the four group rats **(B)** The rate of *p*-ERK1/2/GAPDH **(C)** The rate of *p*-SGK1/GAPDH **(D)** The rate of NHERF-1/GAPDH **(E)** The rate of NaPi-2a/GAPDH. The data are expressed as mean ± S.D. *p < 0.05, **p < 0.01.

ERK1/2-SGK1-NHERF-1-NaPi-2a pathway at the downstream of FGF23-Klotho signaling axis plays an important role in phosphorus reabsorption at proximal renal tubular epithelial cells of CKD–MBD. The main signaling molecules of ERK1/2-SGK1-NHERF-1-NaPi-2a pathway include *p*-ERK1/2, *p*-SGK1, NHERF-1 and NaPi-2a (Erben and Andrukhova, 2017). Therefore, we observed the effects of FPS and CTR on the protein expression levels of *p*-ERK1/2, *p*-SGK1, NHERF-1 and NaPi-2a in the kidneys of the CKD–MBD model rats by WB analysis. As shown in Figure 7A, after induction of renal injury, the down-regulated protein expression levels of *p*-ERK1/2 and *p*-SGK1, and the up-regulated protein expression levels of NHERF-1 and NaPi-2a in the kidneys of the CKD–MBD model rats were detected significantly, compared to those of the Sham group rats. After the treatment of FPS or CTR for 3 weeks, the changed protein expression levels of *p*-ERK1/2, *p*-SGK1, NHERF-1 and NaPi-2a in the kidneys of the CKD–MBD model rats were ameliorated obviously, compared to those of the CKD–MBD group rats (Figures 7B–E).

**FIGURE 8:**
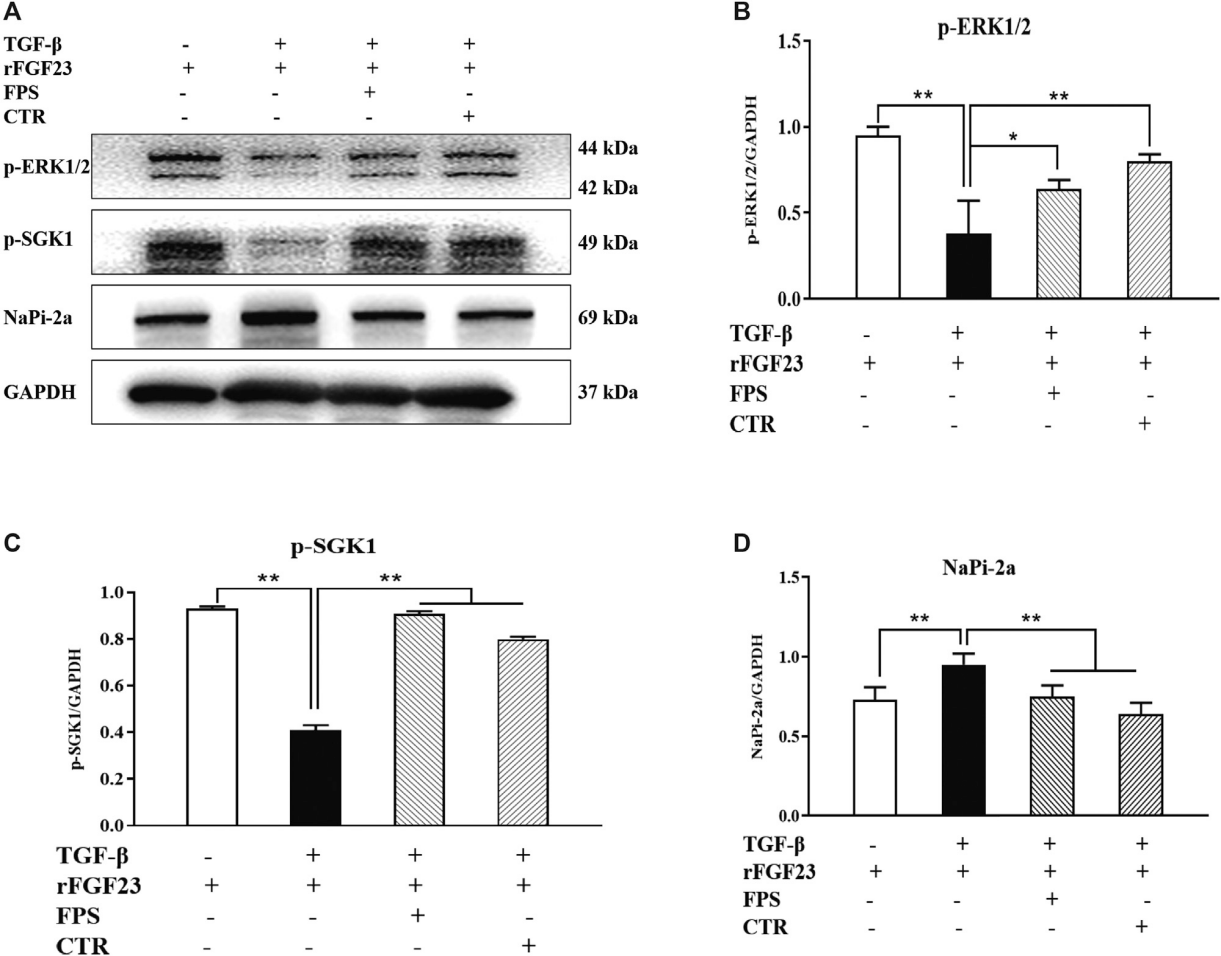
FPS and CTR adjusted ERK1/2-SGK1-NHERF-1-NaPi-2a pathway *in vitro* **(A)** The protein expressional levels of *p*-ERK1/2, *p*-SGK1 and NaPi-2a in the cultured NRK-52E cells exposed to TGF-β and rFGF23 **(B)** The rate of *p*-ERK1/2/GAPDH **(C)** The rate of *p*-SGK1/GAPDH **(D)** The rate of NaPi-2a/GAPDH. The data are expressed as mean ± S.D. *p < 0.05, **p < 0.01.

Likewise, to confirm whether FPS and CTR could influence the expressions of main signaling molecules in ERK1/2-SGK1-NHERF-1-NaPi-2a pathway *in vitro*, we further examined the protein expression levels of *p*-ERK, *p*-SGK1 and NaPi-2a in the cultured NRK-52E cells exposed to TGF-β and rFGF23 for 24 h, compared to the control cells. In Figure 8, our results showed that rFGF23 decreased the protein expression levels of *p*-ERK and *p*-SGK1, and increased the protein expression level of NaPi-2a in the cultured NRK-52E cells exposed to TGF-β, compared to those of the control cells (Figures 8B–D). Furthermore, notably, the treatment with CTR or FPS at the suitable doses for 24 h improved rFGF23-induced changes in the protein expression levels of *p*-ERK, *p*-SGK1 and NaPi-2a in the cultured NRK-52E cells exposed to TGF-β significantly, compared to the treatment with rFGF23. But, ameliorative effects of FPS and CTR on the protein expressions of *p*-ERK, *p*-SGK1 and NaPi-2a were not different obviously (Figures 8B–D).

These results indicated that ERK1/2-SGK1-NHERF-1-NaPi-2a pathway in the kidney could be modulated by FPS and CTR *in vivo* and *in vitro*.

### The Targeted Effects of FPS on Klotho Above ERK1/2-SGK1-NHERF-1-NaPi-2a Pathway *in Vitro*

**FIGURE 9:**
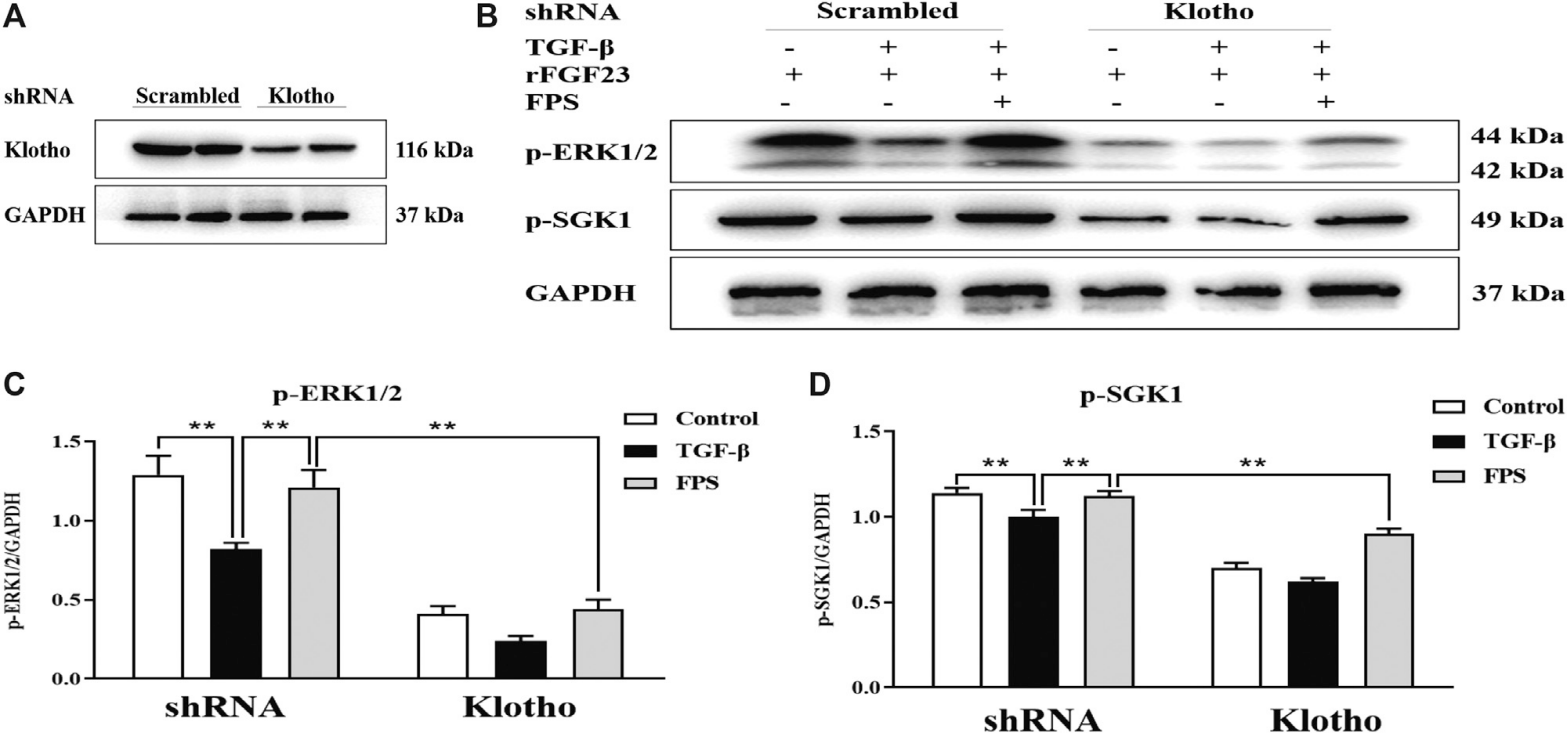
FPS activated ERK1/2-SGK1-NHERF-1-NaPi-2a pathway accurately through Klotho loss reversal *in vitro* **(A)** The protein expressional level of Klotho in the cultured NRK-52E cells transfected by the shRNA-Klotho plasmid **(B)** The protein expressional levels of *p*-ERK1/2 and *p*-SGK1 in the cultured NRK-52E cells exposed to TGF-β and rFGF23 **(C)** The rate of *p*-ERK1/2/GAPDH **(D)** The rate of *p*-SGK1/GAPDH. The data are expressed as mean ± S.D. **p < 0.01.

Klotho is an important upstream factor above ERK1/2-SGK1-NHERF-1-NaPi-2a pathway (Olauson et al., 2014). Thus, to assess whether reversal of Klotho loss by FPS could contribute to activation of ERK1/2-SGK1-NHERF-1-NaPi-2a pathway *in vitro*, we detected impact of Klotho knockdown on FPS improvement of the protein expression levels of *p*-ERK1/2 and *p*-SGK1 as the key signaling molecules in ERK1/2-SGK1-NHERF-1-NaPi-2a pathway activation in the cultured NRK-52E cells exposed to TGF-β and rFGF23 for 24 h, compared to the control plasmid-transfected cells. Here, we constructed a short hairpin RNA (shRNA) plasmid, effectively reducing Klotho protein expression level when transfected into the NRK-52E cells, and then performed the experiment with a control plasmid and an shRNA plasmid specific for Klotho in the NRK-52E cells (Figure 9A). The results showed that the shRNA-Klotho plasmid-transfected cells exhibited decreased basal levels of *p*-ERK1/2 and *p*-SGK1, compared to those in the control plasmid-transfected cells, indicating that Klotho could control their basal expression. Although FPS significantly alleviated TGF-β-induced abnormal expressions of *p*-ERK1/2 and *p*-SGK1 in the control plasmid-transfected cells, these beneficial effects were largely abolished when Klotho was knocked down by shRNA (Figures 9B–D).

These results indicated that ERK1/2-SGK1-NHERF-1-NaPi-2a pathway could be activated by FPS accurately through Klotho loss reversal *in vitro*.

## Discussion

It is reported that CKD–MBD has been one of common and frequent complications of CKD patients in the middle and advanced stages (Hocher and Pasch, 2017; Liu et al., 2018). To explain its pathogenic and therapeutic mechanisms, several experimental models of CKD–MBD including the 5/6 nephrectomized rat model have been widely used ever since. Despite this, it is commonly known that it is difficult to induce typical bone abnormality and vascular calcification in the 5/6 nephrectomized rat model (Shobeiri et al., 2010). Whereupon, recently, researchers have been developing alternative animal models including the adenine-induced CKD–MBD rat model, which can be considered to exhibit severe bone abnormality and vascular calcification without traumatic renal operations (Hocher et al., 2017). Tamagaki et al. reported that, at 6 weeks after establishing the CKD–MBD rat model induced by feeding 0.75% adenine-containing diet, increased levels of Scr and serum P^4+^ and decreased levels of serum Ca^2+^ and 1,25(OH)_2_D_3_ are both confirmed in all adenine-fed rats (Tamagaki et al., 2006). Besides, increased bone resorption with fibrosis in cortical femur bone, decreased bone resorption and increased bone volume with a larger amount of osteoid in trabecular femur bone are certified, respectively. Correspondingly, in this study, we firstly tried to establish the modified CKD–MBD rat model induced by uninephrectomy and adenine administration with a dose of 150 mg/kg/d. Our results showed that, at 3 weeks after induction of renal injury, renal dysfunction, renal tubulointerstitial damage, calcium-phosphorus metabolic disorder and bone abnormality were sequentially revealed in this CKD–MBD rat model. Hence, we considered that the modified CKD–MBD rat model should be helpful in unraveling renal injury and bone abnormality, and finding the novel therapeutic drugs in human CKD–MBD.

Zhang et al. reported that, in the subtotal nephrectomy CRF model and the cryoinjury-induced CRF model, FPS could decrease elevated levels of Scr and BUN, and attenuate renal histopathological lesion in a dose-dependent manner (Zhang et al., 2003). FPS, a natural compound of *Laminaria japonica*, at the low dose of 200 mg/kg/d, has renoprotective effects that are similar to dexamethasone at the dose of 0.07 mg/kg/d. By comparison, in this study, using the modified CKD–MBD rat model, we further expounded whether FPS and CTR could improve renal injuries within 3 weeks after induction of renal injury. Our data indicated that FPS significantly decreased the levels of UA, Scr and BUN, and reduced the scores of ECM accumulation and collagen deposition in the tubulointerstitial area of the CKD–MBD model rats. Furthermore, the effects of FPS improving Scr and BUN levels were better than CTR. Thus, we confirmed that FPS, superior to CTR, could alleviate renal dysfunction and renal tubulointerstitial damage *in vivo*, which are the main renal injurious characteristics of CKD–MBD.

To our knowledge, in addition to vascular calcification, bone abnormality as another important characteristic of CKD–MBD includes calcium-phosphorus metabolic disorder and osteoporosis-like pathological lesions, which are involved in the unbalance of urine Ca^2+^ and P^4+^, serum Ca^2+^, P^4+^, ALP, FGF23, VD_3_ and iPTH, as well as BMD (Tamagaki et al., 2006). In this study, we found that the levels of urine Ca^2+^ and P^4+^, serum Ca^2+^, P^4+^ and VD_3_, as well as osteoporosis-like pathological lesions showed by histopathological observation and micro-CT analysis in the femur bone of the CKD–MBD model rats were both ameliorated significantly after the treatment of FPS or CTR for 3 weeks. Here, unfortunately, beneficial changes of serum FGF23, BMD and iPTH (shown in Supplemental Figure 3) in the CKD–MBD model rats treated by FPS or CTR within 3 weeks could not be detected clearly. Interestingly, different from our results, Hwang et al. recently reported that the treatment of FPS at the dose of 280 mg/kg/d plus calcium carbonate could raise BMD and bone ash weight in 40 weeks old C57BL/6j female mice within 4 weeks (Hwang et al., 2016); Besides, the level of serum FGF23 (circulating FGF23) can be decreased by the treatment of metformin for 8 weeks in the same CKD–MBD rat model-induced by adenine administration (Neven et al., 2018). Anyway, we had reasons to believe that FPS and CTR could improve calcium-phosphorus metabolic disorder and bone lesion in the CKD–MBD model rats. But, the direct target of FPS *in vivo* is neither FGF23 nor iPTH.

Recent advances in FGF23-Klotho signaling axis in the kidney have shown that, at proximal renal tubular epithelial cells, FGF23 as a bone-derived hormone suppresses phosphorus reabsorption by a Klotho dependent activation of ERK1/2 and SGK1, leading to the phosphorylation of NHERF-1 and subsequent internalization and degradation of NaPi-2a. Moreover, at proximal and distal renal tubular epithelial cells, FGFR1 is probably the dominant FGF receptor and mediates regulative effects of FGF23 by forming a complex with membrane-bound Klotho in the basolateral membrane (Déliot et al., 2005; Lederer, 2014). We thereby proposed that regulating FGF23-Klotho signaling axis and its downstream ERK1/2-SGK1-NHERF-1-NaPi-2a pathway in the kidney is a successful way to identify therapeutic mechanisms *in vivo* and *in vitro* of FPS on attenuating phosphorus reabsorption. Our data in this study clearly indicated that decreased expressions of Klotho, *p*-ERK1/2 and SGK1 and increased expressions of FGFR1, NHERF-1 and NaPi-2a in the kidneys of the CKD–MBD model rats and the cultured NRK-52E cells exposed to TGF-β and rFGF23 were obviously revealed, respectively, in the meantime, concomitant with increased serum P^4+^ (hyperphosphatemia), decreased urine P^4+^, increased serum FGF23 and osteoporosis-like pathological lesions in the femur bone. These results forcefully suggested that FGF23-Klotho signaling axis and its downstream ERK1/2-SGK1-NHERF-1-NaPi-2a pathway in the kidney were both dysregulated in the CKD–MBD rat model, and there was a strong causality between unbalanced signaling pathways and phosphorus reabsorption. More importantly, we firstly found that FPS, similar to CTR, could not only significantly ameliorate phosphorus reabsorption, but also simultaneously regulate FGF23-Klotho signaling axis and ERK1/2-SGK1-NHERF-1-NaPi-2a pathway in the kidney *in vivo* and *in vitro*.

Here, targeting FGF23-Klotho signaling axis and its downstream ERK1/2-SGK1-NHERF-1-NaPi-2a pathway, how to understand accurate target of FPS adjusting phosphorus reabsorption? It is reported that, the cleaved Klotho directly regulates calcium and phosphorus excretion in the kidney, participates in systemic mineral homeostasis by 1-α hydroxylase activity, PTH and FGF23 secretion, and leads to the activation of ERK1/2-SGK1-NHERF-1-NaPi-2a pathway at upstream (Sakan et al., 2014). Therefore, we further supposed that the effects of FPS improving phosphorus reabsorption might be targeted to Klotho. Our data in this study distinctly indicated that reduced protein expression levels of Klotho *in vivo* and *in vitro* could be recovered by FPS significantly, and that, reversal of Klotho loss by FPS accurately could contribute to activation of ERK1/2-SGK1-NHERF-1-NaPi-2a pathway in the cultured NRK-52E cells transfected by the shRNA-Klotho plasmid.

Finally, we need to bring up some additional points. Firstly, why the 880 mg/kg/d dose of FPS is only chosen in this study? Our data in preliminary experiment showed that the 880 mg/kg/d dose of FPS could attenuate renal fibrosis significantly *in vivo*, and decrease the levels of ALT and AST in this CKD–MBD rat model (shown in Supplemental Figure 4). In addition, FPS at the 880 mg/kg/d dose had no negative effects on histopathological morphology of the liver *in vivo* (data not shown). Secondly, whether vascular calcification can be treated by FPS in this CKD–MBD rat model? No doubt this model is prevalently used for further comprehensive analyses of vascular calcification in CKD, but it has some inevitable issues in terms of demonstrating therapeutic mechanisms of FPS on vascular calcification such as reversible hyperuricemia and systemic inflammation induced by adenine. Unfortunately, in this study, we have no enough data on FPS treatment to address these problems (data not shown). Thirdly, what are potential mechanisms of FPS regulating calcium reabsorption? In distal renal tubules, circulating FGF23 binds to FGFR-Klotho receptor complex, and activates ERK1/2, SGK1 and with-no-lysine kinase1/4 (WNK1/4) complex. Activation of WNK signaling increases luminal membrane abundance of glycosylated transient receptor potential vanilloid-5 (TRPV5) and sodium-chloride cotransporter (NCC), leading to increased calcium and sodium reabsorption in distal renal tubular (Lambers et al., 2006; Jiang et al., 2007; Jiang et al., 2008; McCormick et al., 2008; Cha et al., 2010). Obviously, much more work in the future needs to be done to understand comprehensive therapeutic mechanisms of FPS by which FGF23-Klotho signaling axis and ERK1/2-SGK1-WNK1/4-TRPV5 pathway both control calcium reabsorption.

## Conclusion

**FIGURE 10:**
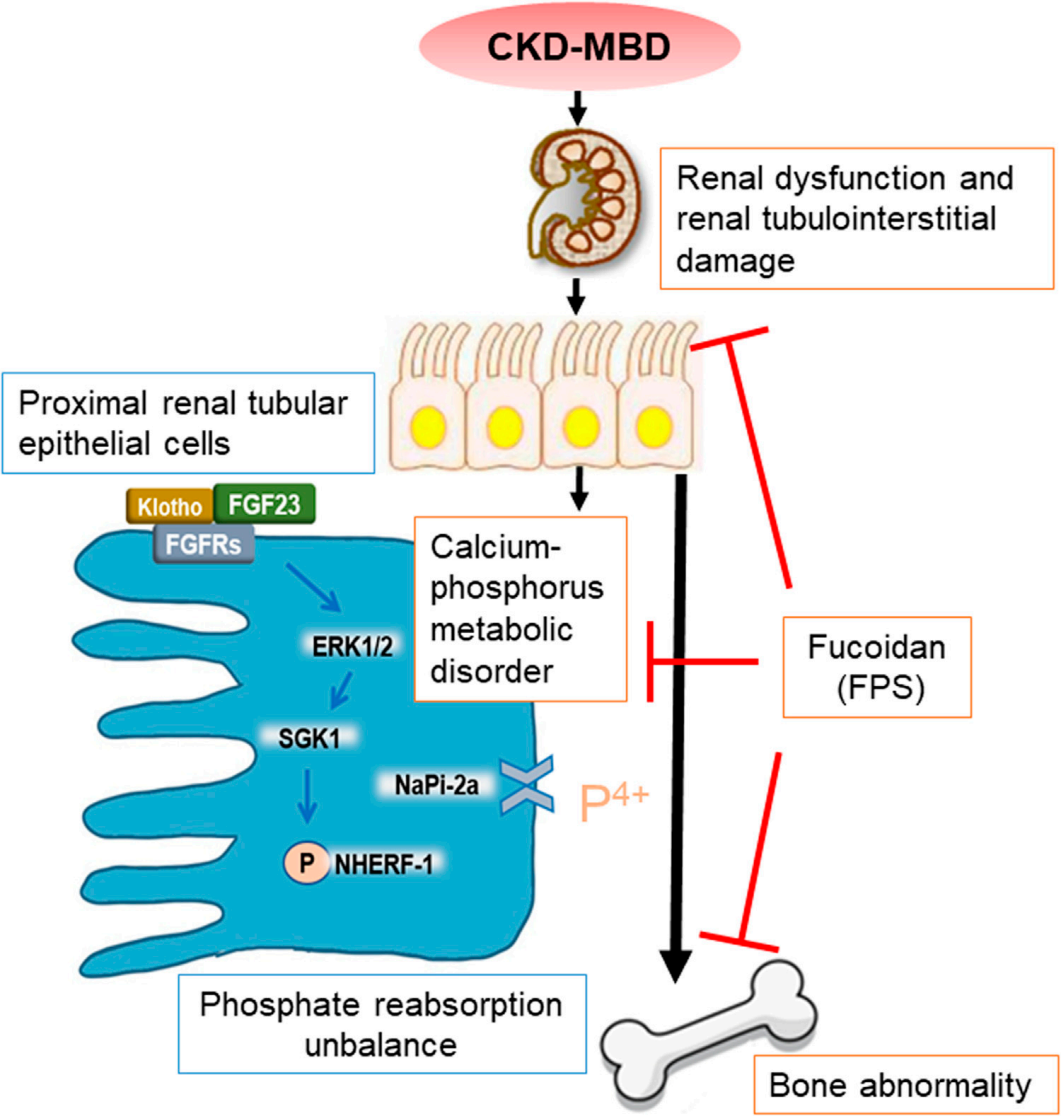
Overview of FPS ameliorating renal injury-related calcium-phosphorus metabolic disorder and bone abnormality in the CKD–MBD model rats.

In summary, in this study, we emphatically demonstrated that FPS, a natural anti-renal dysfunction drug, similar to CTR, improves renal injury-related calcium-phosphorus metabolic disorder and bone abnormality in the CKD–MBD model rats. More importantly, we firstly found that beneficial effects *in vivo* and *in vitro* of FPS on phosphorus reabsorption are closely associated with regulation of FGF23-Klotho signaling axis and ERK1/2-SGK1-NHERF-1-NaPi-2a pathway in the kidney (Figure 10). This study provided pharmacological evidences that FPS directly contributes to the treatment of CKD–MBD.

## Data Availability Statement

The original contributions presented in the study are included in the article/Supplementary Material, further inquiries can be directed to the corresponding authors.

## Ethics Statement

The animal study was reviewed and approved by The Animal Ethics Committee of Nanjing University Medical School.

## Author Contributions

Y-GW, G-WW, and F-LC provided the conception and design of research. B-HL, C-CY, Y-LL, H-MY, W-WW, Q-JF, WW, and M-ZW performed the experiments; B-HL, C-CY, and Z-YW analyzed the data and interpreted the results of experiments; B-HL and YT prepared the figures; B-HL, G-WW, and Y-GW drafted the manuscript; F-LC and C-CY edited and revised the manuscript; Y-GW and B-HL approved the final version of manuscript.

## Funding

This work was supported by two grants from the National Natural Science Foundation of China (81573903 and 81603675), a grant from the Natural Science Foundation of Jiangsu Province for Young Scholars (BK20161046), a grant from the Priority Academic Program Development of Jiangsu Higher Education Institutions (PAPD), a grant from the Open Projects of the Discipline of Chinese Medicine of Nanjing University of Chinese Medicine Supported by the Subject of Academic priority discipline of Jiangsu Higher Education Institutions (NO.ZYX03KF016), a grant from Nanjing Medical Science and Technique Development Foundation (QRX17042) and a grant from Nanjing Famous TCM Doctor’s Studio Programme.

## Conflict of Interest

G-WW was employed by the company Jilin Province Huinan Chonglong Bio-Pharmacy Co., Ltd.

The remaining authors declare that the research was conducted in the absence of any commercial or financial relationships that could be construed as a potential conflict of interest.

## Glossary

ALP: alkaline phosphatase
ALT: Alanine aminotransferase
AST: Aspartate aminotransferase
BMD: bone mineral density
BUN: blood urea nitrogen
BV: bone volume
BV/TV: bone volume/tissue volume ratio
BW: body weight
CKD: chronic kidney disease
CKD–MBD: chronic kidney disease-mineral and bone disorder
ECM: extracellular matrix
ERK1/2: extracellular signal-regulated kinase1/2
FBBMD: femur bone mineral density
FGF23: fibroblast growth factor 23
FGFR1: FGF receptor1
GAPDH: glyceraldehyde-3-phosphate dehydrogenase
HE: Hematoxylin-Eosin
IHC: immunohistochemistry
iPTH: intact parathyroid hormone
KHI: kidney hypertrophy index
KW: kidney weight
LM: light microscopy
NaPi-2a: sodium-phosphate co-transporter-2a
NF: nuclear factor
NHERF-1: Na^+^/H^+^ exchange regulatory cofactor-1
PAS: periodic acid-Schiff
p-ERK1/2: phosphorylated ERK1/2
rFGF23: recombinant human FGF23 R176/179Q
Scr: serum creatinine
SGK1: serum/glucocorticoid-regulated kinase1
shRNA: specific short hairpin RNA
TBBMD: total bone mineral density
Tb.N: trabecular number
Tb.Sp: trabecular bone separation
Tb.Th: trabecular bone thickness
TGF-β: transforming growth factor-β
TV: total VOI volume
UA: uric acid
VD3: 25-hydroxyvitamin D3
WB: Western blotting
