# Corrigendum: Fucoidan ameliorates renal injury-related calcium-phosphorus metabolic disorder and bone abnormality in the CKD–MBD model rats by targeting FGF23-Klotho signaling axis

**DOI:** 10.3389/fphar.2024.1446609

**Published:** 2024-07-23

**Authors:** Bu-Hui Liu, Fee-Lan Chong, Can-Can Yuan, Ying-Lu Liu, Hai-Ming Yang, Wen-Wen Wang, Qi-Jun Fang, Wei Wu, Mei-Zi Wang, Yue Tu, Zi-Yue Wan, Yi-Gang Wan, Guo-Wen Wu

**Affiliations:** ^1^ Department of Traditional Chinese Medicine, Nanjing Drum Tower Hospital Clinical College of Traditional Chinese and Western Medicine, Nanjing University of Chinese Medicine, Nanjing, China; ^2^ Nephrology Division, Affiliated Hospital of Nanjing University of Chinese Medicine, Nanjing, China; ^3^ The School of Pharmacy, Management and Science University, Shah Alam, Malaysia; ^4^ Department of Traditional Chinese Medicine, Nanjing Drum Tower Hospital, The Affiliated Hospital of Nanjing University Medical School, Nanjing, China; ^5^ Department of Traditional Chinese Medicine Health Preservation, Acupuncture, Moxibustion and Massage College, Health Preservation and Rehabilitation College, Nanjing University of Chinese Medicine, Nanjing, China; ^6^ Department of Social Work, Meiji Gakuin University, Tokyo, Japan; ^7^ Jilin Province Huinan Chonglong Bio-Pharmacy Co., Ltd., Huinan, China

**Keywords:** fucoidan, chronic kidney disease-mineral and bone disorder, FGF23-klotho signaling axis, phosphorus reabsorption, ERK1/2-SGK1-NHERF-1-NaPi-2a pathway

In the published article, there was an error in Figure 5 as published. One image was erroneously overlapped in Figure 5A -FGF23-Sham + Vehicle, and in Figure 5A -FGF23-CKD-MBD + CTR. The corrected Figure 5 and its caption appear below.

**FIGURE 5:**
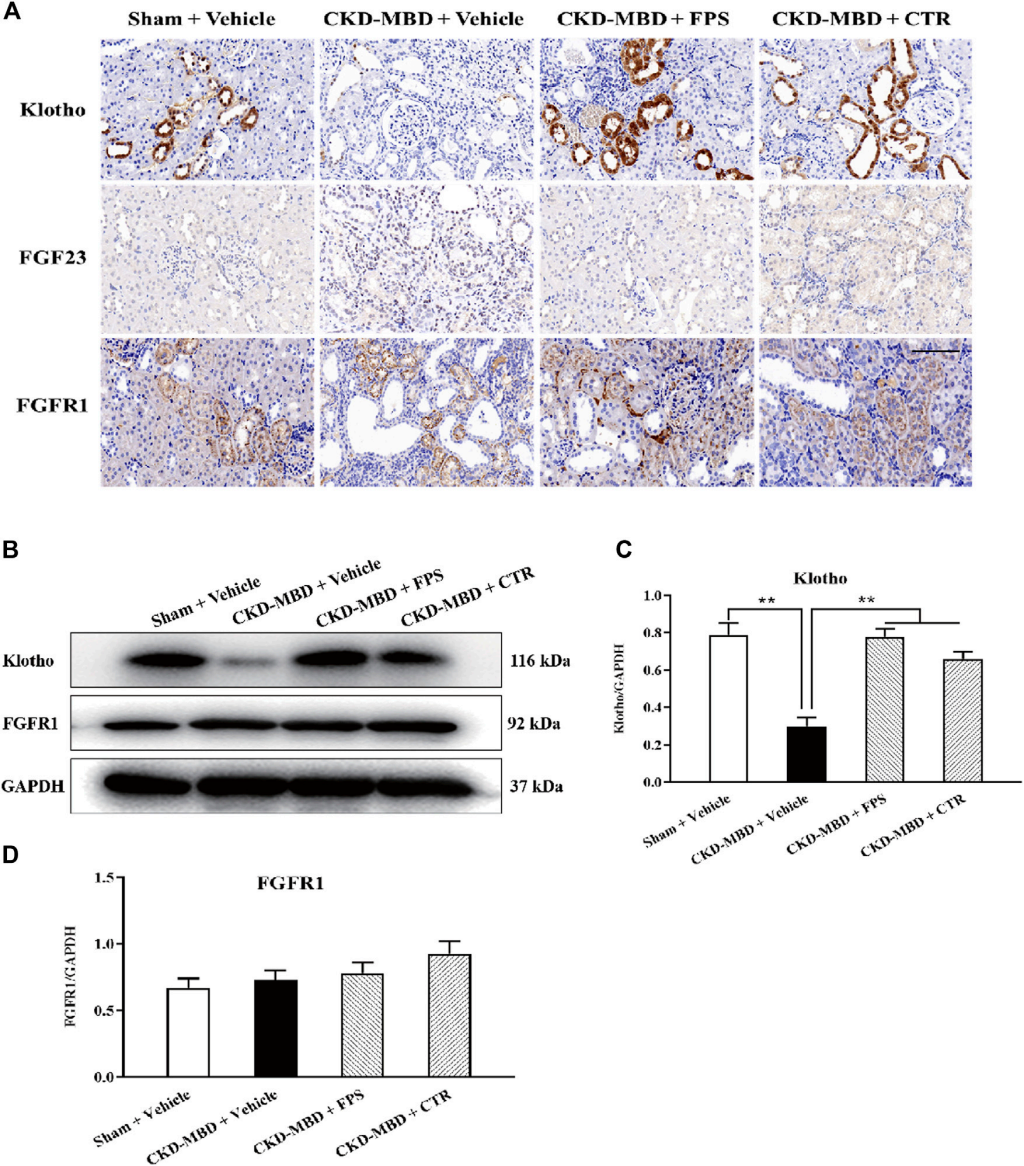
FPS and CTR regulated FGF23-Klotho signaling axis in vivo **(A)** The protein expressional characteristics of Klotho, FGF23 and FGFR1 in renal tubulointerstitium of the kidneys in the four group rats. IHC staining × 200. Scale bar 100 µm **(B)** The protein expressional levels of Klotho, FGF23 and FGFR1 in the kidney of the four group rats **(C)** The rate of Klotho/GAPDH **(D)** The rate of FGF23/GAPDH **(E)** The rate of FGFR1/GAPDH. The data are expressed as mean ± S.D. **p < 0.01.

The authors apologize for this error and state that this does not change the scientific conclusions of the article in any way. The original article has been updated.

