# Supplementary figures and images for: Fucoidan Ameliorates Renal Injury-Related Calcium-Phosphorus Metabolic Disorder and Bone Abnormality in the CKD–MBD Model Rats by Targeting FGF23-Klotho Signaling Axis
Source: Front Pharmacol. 2021 Jan 28;11:586725. doi: 10.3389/fphar.2020.586725 (PMC7941278; doi:10.3389/fphar.2020.586725)

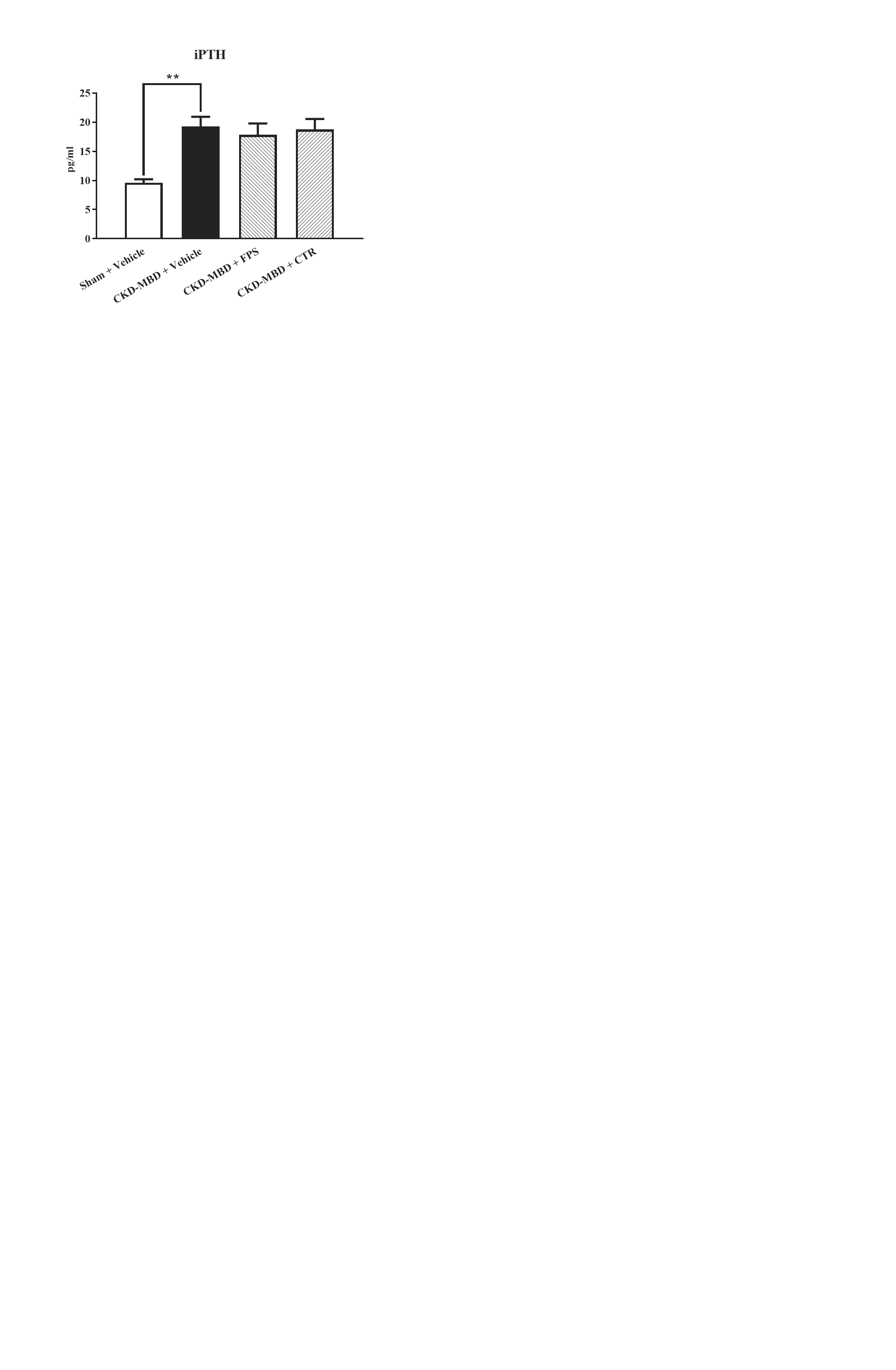

Supplement: Supplementary file 1 [file Image3.TIFF]

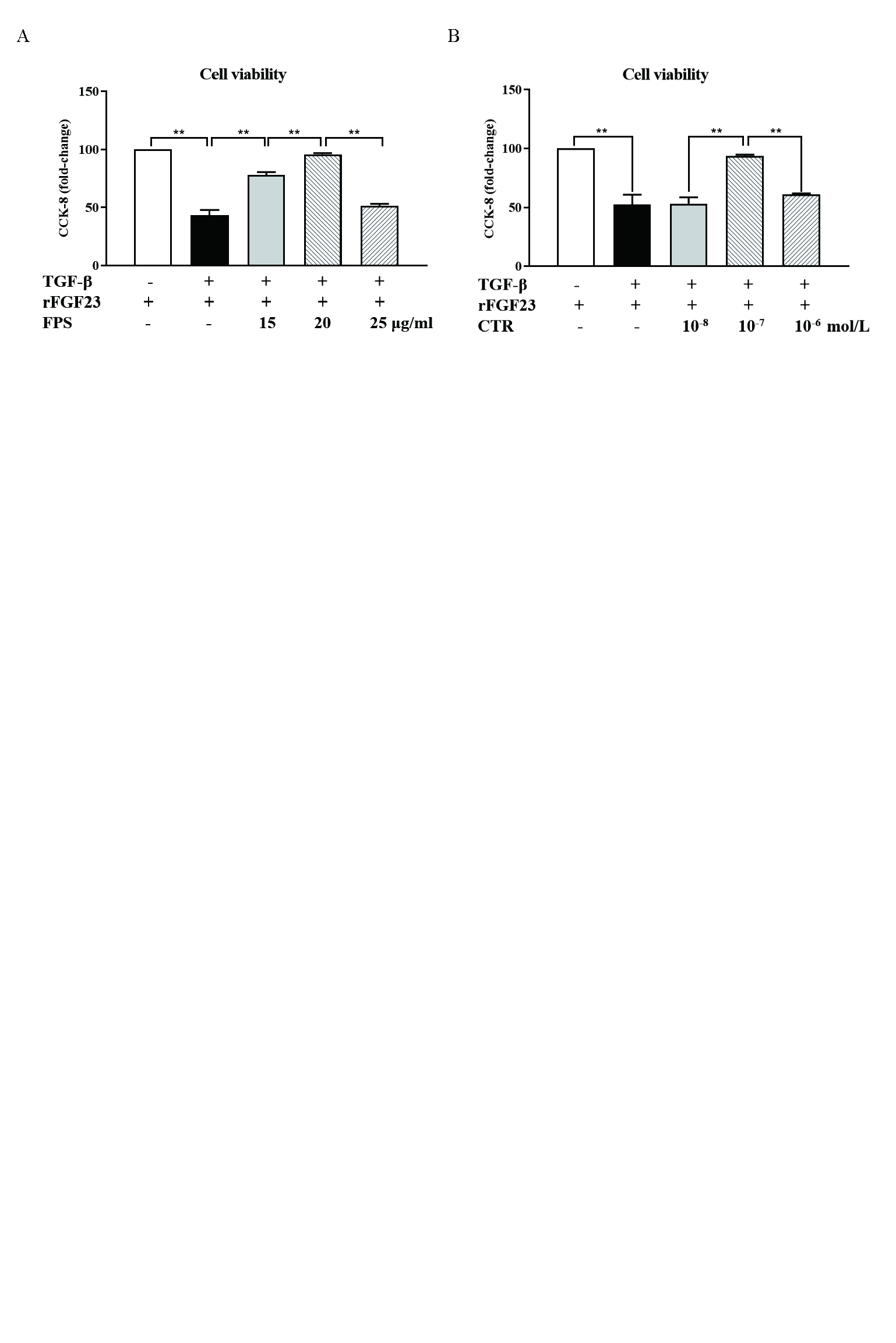

Supplement: Supplementary file 2 [file Image2.TIF]

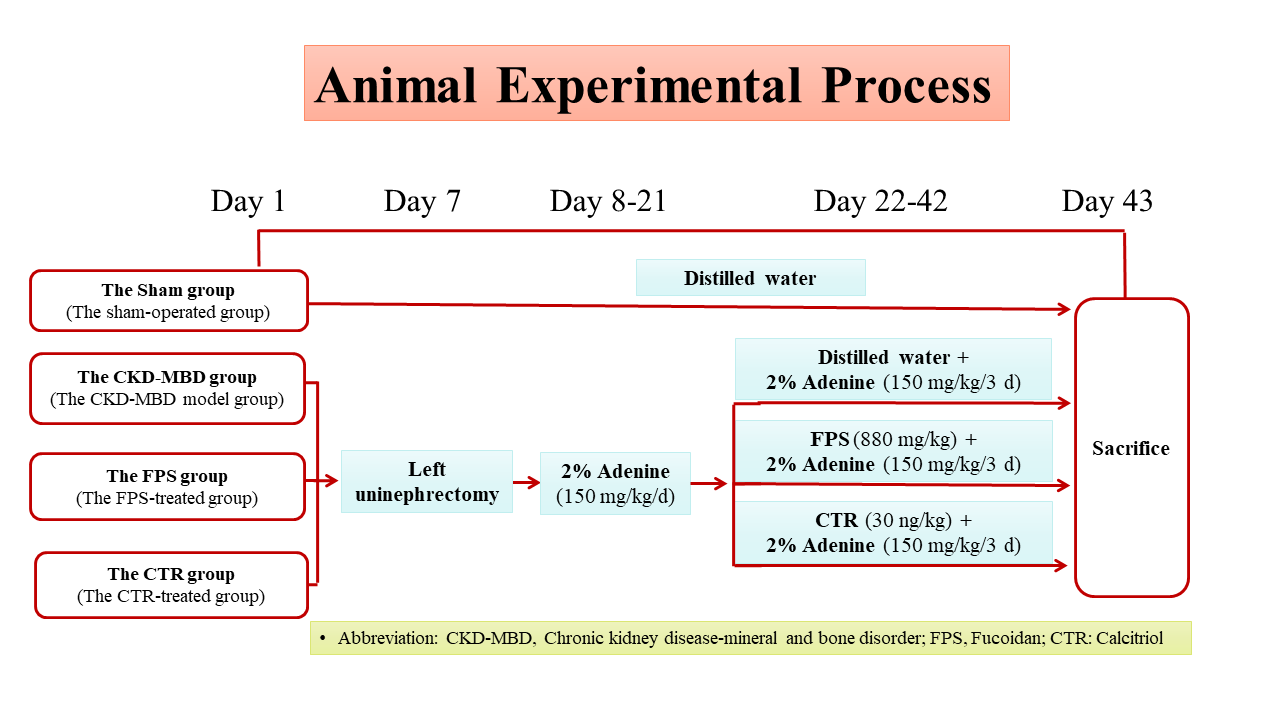

Supplement: Supplementary file 3 [file Image1.TIF]
